# An Overview of Mycorrhiza in Pines: Research, Species, and Applications

**DOI:** 10.3390/plants13040506

**Published:** 2024-02-11

**Authors:** Valentyna Dyshko, Dorota Hilszczańska, Kateryna Davydenko, Slavica Matić, W. Keith Moser, Piotr Borowik, Tomasz Oszako

**Affiliations:** 1 Ukrainian Research Institute of Forestry and Forest Melioration Named after G. M. Vysotsky, 61024 Kharkiv, Ukraine; valya_dishko@ukr.net (V.D.); kateryna.davydenko74@gmail.com (K.D.); 2 Department of Forest Ecology, Forest Research Institute, Sękocin Stary, Braci Leśnej 3, 05-090 Raszyn, Poland; d.hilszczanska@ibles.waw.pl; 3 Department of Forest Mycology and Plant Pathology, Uppsala BioCenter, Swedish University of Agricultural Sciences, P.O. Box 7026, 75007 Uppsala, Sweden; 4 Institute for Sustainable Plant Protection (IPSP), National Research Council of Italy (CNR), Strada delle Cacce 73, 10135 Torino, Italy; slavica.matic@ipsp.cnr.it; 5 US Department of Agriculture, Forest Service, Rocky Mountain Research Station, 2500 S. Pine Knoll Dr., Flagstaff, AZ 86001, USA; warren.k.moser@usda.gov; 6 Faculty of Civil Engineering and Environmental Sciences, Bialystok University of Technology, Wiejska 45E, 15-351 Białystok, Poland; pborow@poczta.onet.pl

**Keywords:** mushroom–root symbiosis, pathogens, artificial mycorrhization, forest protection, climatic changes

## Abstract

In the latest literature, climate models show that the conditions for pines, spruces, larches, and birches will deteriorate significantly. In Poland, as well as in other European countries, there are already signs of the decline of these species. This review article deals with the symbiotic relationships between fungi and plants, which can hardly be overestimated, using the example of pine trees. These are the oldest known symbiotic relationships, which are of great benefit to both components and can help plants, in particular, survive periods of severe drought and the attack of pathogens on the roots. This article describes symbioses and their causal conditions, as well as the mycorrhizal components of pine trees and their properties; characterizes ectomycorrhizal fungi and their mushroom-forming properties; and provides examples of the cultivation of pure fungal cultures, with particular attention to the specificity of the mycorrhizal structure and its effects on the growth and development of *Pinus* species. Finally, the role of mycorrhiza in plant protection and pathogen control is described.

## 1. Introduction

A refined model of the predicted distributions of 12 European forest tree species under three climate change scenarios from 2061 to 2080 showed that *Abies alba*, *Fagus sylvatica*, *Fraxinus excelsior*, *Quercus robur*, and *Quercus petraea* would be among the ‘winners’, while the ‘losers’ would be the pioneer species *Betula pendula*, *Larix decidua*, *Picea abies*, and *Pinus sylvestris* [1].

Assuming limited migration, most of the species analyzed would be confronted with a considerable reduction in the areas of suitable habitat. The ecological consequences of the predicted shrinking of distribution areas would be serious for both forestry and nature conservation. Other studies on the effects of climate change on Scots pine growth across Europe confirm this tendency [2]. If these predictions turned out to be true 75% of forest-forming species in Poland would be affected.

Mycorrhiza is one of the fundamental phenomena of nature, characteristic of land plants from the moment of their formation, representing a form of symbiosis between plants, fungi, and bacteria. More than 90% of vascular plants in underground ecosystems are associated with mycorrhizal fungi, which have direct access to the assimilates of their hosts and serve as carriers of mineral nutrients [3,4].

Mycorrhizal fungi serve as mediators in the interactions of plants with various soil microbes and influence the dynamics with pathogens as well as with the mutualists of the mycorrhizosphere, which contribute to vitamin production and protect against antagonists [5,6,7]. The symbiotic relationship goes even further, as mycorrhizal root connections influence underground plant properties, regulate relationships between plants, and alter general ecosystem processes.

Mycorrhizal networks, which extend over extensive underground connections, establish physical links between plants of the same or different species. This network facilitates the transfer of nutrients and the transmission of chemical signals between plants [8]. The different types of mycorrhiza—arbuscular mycorrhiza (AM), ectomycorrhiza (ECM), ericoid mycorrhiza (ErM), and orchid mycorrhiza (OM)—each have different evolutionary backgrounds, anatomical structures, and ecological functions. Consequently, these mycorrhizal associations exert different influences on plant protection, nutrient acquisition, and the cycling of carbon and nutrients in the soil.

Mycorrhizal fungi generally play a central role in influencing the dynamics of plant populations and communities, with notable differences between different mycorrhizal types. Our synthesis of existing knowledge highlights the impact of mycorrhizal associations on plant interactions and ecological specialization. Our results suggest that mycorrhizal associations themselves, together with fungal diversity and specific mycorrhizal types, have direct or indirect effects on plant dispersal and competition. Together, these factors shape plant populations and communities and determine the coexistence and diversity of plants at the local level [9].

Mycorrhizal associations are of great benefit to terrestrial plants, as they improve access to nutrients and stress tolerance. These fungi mediate plant interactions with the soil microbiome and influence nutrient uptake, vitamin production, and protection against pathogens [7]. Given the importance of symbiotic relationships between plants and mycorrhizal fungi, the use of mycorrhizal fungi is important for rehabilitating degraded soils and increasing their fertility [10,11].

## 2. Invaluable Mycorrhizal Network

The phenomenon of symbiosis has long been at the center of scientific interest and is also of interest to representatives of various biological disciplines. Scientists are interested in the forms and types of mycorrhizal fungi, their distribution in the plant and animal world, the nature of the relationships between the components of symbiotic associations, and the adaptations of symbionts [8].

It is widely known that the network of mycorrhizal fungi in the soil, which connects the root systems of different plants, is divided according to mycorrhizal type and association specificity and influences the redistribution of carbon and nutrients, underground signaling, and the regulation of competition. Mycorrhizal fungi transport plant carbon into the soil to maintain the microbiome of the mycorrhizosphere. Larger plants significantly contribute to the maintenance of mycorrhizal networks in certain symbioses.

There is also nutrient transfer between plants, but indirect pathways such as the decomposition of roots and leaf litter often play a more important role than transport by mycorrhizae. Mycorrhizal fungi communicate with plants via various compounds that mediate underground signaling and kin recognition. Signaling and nutrient transfer are most pronounced between related plants, suggesting direct communication [12]. Communication between unrelated plants and plants of different mycorrhizal types is weaker, apart from occasional losses due to parasitism.

In forest communities, an important factor for species diversity is the dependence on the density of conspecifics. However, as an important biotic component of the soil environment, soil microbes can also contribute to the formation of plant diversity and biomass patterns [12,13]. The rich diversity and biomass of ericoid, ectomycorrhizal, and saprotrophic fungal guilds in forest soils play a crucial role in conservation and ecosystem processes. In addition, many ectomycorrhizal fungi are important for nature conservation. However, there is a lack of comprehensive information on the functions of soil fungi, their relationships to forest conservation values, and the effects of inter- and intra-guild interactions on soil organic matter.

Trees generally form two main types of mycorrhizal associations: arbuscular mycorrhizal (AM) associations with fungi from the subphylum Glomeromycota and ectomycorrhizal (EM) associations with fungi belonging mainly to the phyla Ascomycota and Basidiomycota [14]. An increasing number of studies have focused on the mycorrhizal network in different countries around the world [3,4,7,14,15,16,17], including Poland [18,19,20,21] and Ukraine [22,23,24]. Pachlewski’s team in Poland carried out the most important mycorrhiza identifications [25], contributing to the physiology of mycorrhiza, the isolation of mycorrhizal fungi and their influence on the vitality of trees, and the artificial mycorrhization of forest tree seedlings under laboratory conditions.

Unfortunately, soils that have been degraded, for example, due to many years of agricultural use, often lack mycorrhizal fungi [10]. Therefore, before planting pine seedlings, it is recommended to introduce organic material from the forest into the soil, e.g., in the form of logging residues. This treatment not only increases the biodiversity of the mycobiome and microbiome [26] but also restores the fungal species that form mycorrhizal associations with pine seedlings, making them healthier and better adapted, for example, to longer periods of drought (as a result of climate change). This means that the silvicultural goal of creating grassy and diverse stands for future generations has a better chance of being realized [11].

## 3. Mycorrhizal Components of Pine Trees and Their Properties

Pinus species are highly dependent on the presence of compatible ectomycorrhizal (ECM) fungi. The presence of mycorrhizal communities in the soil, which are characteristic of this species, provides the trees with access to nutrients in the soil and creates favorable conditions for their growth and development.

Large-scale mycological observations in Poland have identified species of mycorrhizal fungi that accompany pine regardless of the type of forest area and have confirmed the ability of many fungi to form ectomycorrhizae with it [25]. Species from the genera *Amanita*, *Tricholoma*, *Suillus*, *Rhizopogon*, and *Hebeloma* are probably among the most important associates of pine. Genera such as *Lactarius*, *Cortinarius*, and *Russula* contain a wide range of species that can form ectomycorrhizas with pine. The genera *Collybia*, *Clitocybe*, and *Mycena* are demonstrably unable to form mycorrhiza with pine trees [27,28].

Studies conducted in Ukraine have demonstrated symbiotic relationships between pines and the mycorrhizal fungi *Suillus luteus* and *Amanita muscaria* [29]. *Scleroderma* species form symbiotic relationships not only with pine trees but also with *Pinus patula* [30], *P. menziesii*, and *P. pinaster* [31]. Such symbioses were also found in forests with *P. sylvestris*, *P. resinosa*, *Larix decidua* [32], *Betula pendula*, *Quercus petraea/robur* [33,34,35], *Alnus* spp. [36], *Picea abies* [17], and *Eucalyptus* spp. [37]. To date, the genus *Scleroderma* comprises about 60 species, and most of them can form mycorrhiza, although these fungi can also exist as saprotrophs [38,39]. One of the main benefits of symbiotic relationships with *Scleroderma* spp. is the increase in vigor and stress resistance of young plants.

*Imleria badia* (Fr.) is also a mycorrhizal partner of pine. It has an ecological advantage over other pine companions and grows well under various environmental conditions, even in metal-polluted areas [40]. The fungus *Imleria badia* grows in coniferous and mixed forests in Europe, Australia, and Japan and most commonly forms mycorrhizae with pines and spruces [41]. It is widespread in North America (from eastern Canada, western Minnesota, and south to North Carolina), as well as in China and Southeast Asia.

## 4. Peculiarities of Ectomycorrhizal Fungi and Their Characteristics

The symbionts of most temperate and boreal forest trees are ectomycorrhizal (ECM) fungi, which supply their host plants with nutrients and water in exchange for carbon [42,43]. Scientists hypothesize that ECMs oxidize organic matter in the soil, thereby releasing nitrogen and leading to improved plant nutrition, but direct empirical evidence for this is lacking [44,45]. A quantitative field assessment of the uptake of organic nitrogen by ECM fungi would be an important advance in understanding the potential extent of this phenomenon.

Most ectomycorrhizal (ECM) fungi are characterized by the absence of invertase [46,47], which distinguishes them from phytopathogenic [48] and ericaceous mycorrhizal fungi [49]. The ability of plants and root microorganisms to respond to nutrient deficiency was investigated by Calvaruso et al. [50]. They found that plants and their microbial partners can alter their life cycles within limits to maintain an appropriate level of inorganic nutrient availability [51,52].

There are several views that ectomycorrhizal (ECM) fungi differ in their ability to supply their host plants with nitrogen fixed in soil organic matter and that this ability can affect soil carbon storage both positively and negatively [53]. At the same time, it remains unclear whether all ECM taxa can store the nitrogen fixed in soil organic matter [45,54]. Recent studies suggest that ECMs alter organic matter stocks differently in different soil horizons [55], which may be related to differences in the surrounding biotic communities [56]. This remains a largely unexplored ecological aspect that may have important consequences for plant growth, as well as soil carbon and nitrogen cycling.

The ability to accumulate heavy metals and promote the survival and growth of tree species on degraded soils has been studied by many scientists, who have emphasized that the economic efficiency of the application of mycorrhizal fungi in these areas can have long-term results [57,58,59].

Adaptive tolerance to Cd is a rare phenomenon in plants and their symbiotic partners [60,61]. The accumulation of this and other heavy metals in plants impairs the expression of their genes [62], suppresses DNA repair [63], causes a decline in photosynthesis, reduces the uptake of water and nutrients [64,65], and leads to visible symptoms of damage, such as chlorosis, growth inhibition, browning of the root tips, and ultimately death [66].

Krupa and Kozdrój [67] reported on the positive role of ectomycorrhizal fungi and the bacteria associated with the respective fungal species in the distribution of heavy metals in the roots and shoots of inoculated pines (*Pinus sylvestris* L.). They investigated the ability to promote the translocation of Zn (II), Cd (II), and Pb (II) in species such as *Scleroderma citrinum*, *Amanita muscaria*, and *Lactarius rufus* [67,68].

The double inoculation of pine seedlings with ectomycorrhizae and bacteria of the genus *Pseudomonas* helped increase the storage capacity of these metals, especially Zn (II). The effectiveness of this approach to protect plants from heavy metals is recommended for use on soils contaminated with heavy metals [67].

Populations of *S. luteus* are tolerant to soil contamination with heavy metals (zinc, copper, cadmium, etc.) [69,70]. Plants inoculated with *S. luteus* have adapted to cadmium and accumulate more fruiting body biomass than non-adapted plants, representing a kind of barrier that prevents the translocation of heavy metals into the plant tissue [71,72].

Studies carried out in Poland have shown that the concentrations of aluminum (Al), cadmium (Cd), and lead (Pb) in the mycorrhizae formed by the fungal species studied varied greatly, from low to high. The most intensive uptake of cadmium (Cd) was found in the species *Amanita muscaria*, whereas the highest concentration of aluminum (Al) was found in *Thelephora terrestris*. The mycorrhizal accumulation of iron (Fe), manganese (Mn), and zinc (Zn) was not significant, but these metals were generally taken up by most fungi [18].

The results of Cejpková’s study [73] proved the ability to accumulate trace elements in tissues, especially gold (Ag), cadmium (Cd), chlorine (Cl), and zinc (Zn), in species such as *I. badia* and *Thelephora terrestris*. The concentrations of these metals in the ECM tissues were significantly higher than in the plant roots. Scientists hypothesize that ECMs benefit the host by forming a protective barrier against heavy metal toxicity [67].

The species *S. cutrinum* can be regarded as a source of natural melanin. This fungus has broad insecticidal [74], antibacterial [75], antifungal [30,76], and antiviral [77] activity, including radioprotective, thermoregulatory, chemoprotective, antitumor, antiviral, antimicrobial, immunostimulatory, and anti-inflammatory properties [78,79,80]. *Suillus cutrinum* produces cyathin-like antibiotics that inhibit some types of bacteria and fungi [81]. Scientists have identified effective doses (ED50) of DMVA compounds that can significantly inhibit mycelial growth of *Phytophthora palmivora* and *Colletotrichum gloeosporioides* at concentrations of 58 and 81 μg/mL, respectively [76,77].

The ability to produce substances with steroidal character is characteristic of the species *S. citrinum*, which are natural inhibitors against the phytopathogenic fungi *Phytophthora palmivora* and *Colletotrichum gloeosporioides* [82]. The species *I. badia* (Fr.) is rich in antioxidant compounds [83,84]. It contains vitamins, especially those of the B group, as well as macro- and microelements [85], which show antibacterial or antifungal activity. Mycorrhizal fungi belonging to the genus *Suillus*, as well as the species *Gomphidius roseus, Rhizopogon luteolus* (III phase), *Laccaria laccata, Boletus luridus*, and *Cortinarius vibratilis*, intensively process auxins like IAA. The genera *Hebeloma, Tricholoma*, and *Xerocomus* (*Imleria*) and the species *Rhizopogon rubescens* and *Cenococcum graniforme* are characterized by lower productivity of IAA. Auxins were not detected in the mycelium of all analyzed fungi of the genera *Amanita, Lactarius, Russula*, and *Cortinarius* (except *C. vibratilis*), in five *Tricholoma* species, and in *Scleroderma verrucosum, S. aurantium, Collybia butyracea*, and *Mycens pura*. The absence of IAA in the mycelium does not always mean that the fungus is unable to produce these compounds, as some fungal species do not accumulate them in the mycelium but release them into the environment. This has been observed in several *Amanita* species, as well as in *Lactarius rufus*, *Cortinarius armillatus*, and *Hygrophorus hypothejus* [25].

Colpaert et al. [69] compared the effect of *Thelephora terrestris* (Ehr.) Ft., *Suillus bovinus* (L.: Fr.) O. Kuntze, and *Scleroderma citrinum* Pers. on the carbon and nitrogen uptake of mycorrhizal and non-mycorrhizal seedlings of *Pinus sylvestris* L. grown in a semi-hydroponic system with nitrogen as a limiting growth factor. The authors concluded that mycorrhiza influences the above- and below-ground distribution of nitrogen. Specifically, they found that the mycelium of *S. citrinum* retains a significant proportion (32%) of the nitrogen supplied to the plants, thereby significantly reducing its assimilation by the host plants.

The ability of mycorrhizal fungi to synthesize various compounds that contribute to the adaptability and development of the host plant, accumulate and retain heavy metals, and influence the distribution of nitrogen could be important for the creation of pine forests on degraded and polluted soils [10,11,69].

## 5. Cultivation of Pure Cultures

The basis of any mycological work is, first of all, the production of pure fungal cultures. Most cultures are obtained by isolating fruiting body tissue, but in some special cases, basidiospores, rhizomorphs, mycorrhizae, infected wood, or other substrates are used [25]. Obtaining pure cultures depends on many factors, including the age and phenological state of the fruiting bodies, the biological properties of the fungi, the selection of optimal ecological and biochemical growth factors, and other growth conditions.

Research into the isolation of pure fungal cultures involves several steps: the selection of biological objects, preparation of culture media, sowing of fruiting bodies in Petri dishes, visual analysis, transplanting the mycelium to a fresh medium for propagation and maintenance of viability, and obtaining cultures on liquid culture media and solid organic and mineral substrates. The isolation of mycelial cultures is carried out in specially equipped microbiological laboratories. The extraction of mycelium from the tissue of freshly harvested fruiting bodies is a very time-consuming and often ineffective process. Pachlewski and Pachlewska [25] carried out large-scale studies in which they isolated sporophores from 124 species of higher fungi of the class *Basidiomycotes*. They obtained a pure culture of 85 species, corresponding to 68% of the fungi isolated from samples in pine plantations. Almost all species from the genera *Amanita, Suillus, Tricholoma, Collybia, Xerocomus*, and *Lactaris* were obtained in pure cultures.

Similar results, except for the genus *Lactarius*, were also obtained in similar studies [86]. The species *Rhizopogon luteolus, Hygrophorus hypothejus, Hebeloma crustuliniforme, H. mesophaeum, Paxillus involutus, Scleroderma aurantium, S. verucosum*, and *Boletus edulis* were characterized by relatively simple isolation and good development in pure cultures [25]. In the studies by Espenshade [87], the success rate for obtaining pure cultures on an agar medium was only 20%, and in the studies by Oddoux, it was 48% [88]. In the studies by Mynakov et al. [89], the success rate for the recovery of *S. luteus* isolates was 20%, and for *S. bovines*, it was 15%. Similar studies were also carried out by other scientists [90,91]. The difficulty of growing species in pure cultures encourages the investigation of clear requirements for the growth and survival of isolates.

Mycorrhizal fungi differ in their ability to utilize organic and inorganic nitrogen sources for their growth [92]. The agarized culture medium used in selection work for the cultivation of pure cultures is prepared in various ways. The most common method is the use of seasoning agar (SA) [46,47]. For this, 20 g of agar-agar is added to 1 L of wort (an intermediate product in beer production) and stirred until completely dissolved. However, the use of SA for cultivation is not always effective. A comparison of the growth parameters and morphological characteristics of the mycelium of *Paxillus involutus* grown on agarized culture media of 10% wort agar (CA) and Murashige-Skoog (MS) proved the superiority of the latter. On the CA medium, mycelial growth started on the tenth day at the earliest, while some of the explants necrotized and showed no signs of growth. Growth of the mycelium on the MS culture medium was detected on the sixth day. Complete overgrowth of the Petri dishes on MS medium was observed for 28–30 days, and colonies with a convex lenticular shape were formed on SA, but complete overgrowth of the culture medium was not observed. The authors concluded that MS medium is suitable for cultivation and propagation of the dry mass, whereas CA should be used for long-term, uninterrupted storage of the pure culture.

Blaudez et al. [72] investigated the effects of different nitrogen and carbon sources and their concentrations in liquid media on mycelial growth of ectomycorrhizal fungi (*Suillus luteus, Scleroderma citrinum, Laccaria laccata*, and *Tricholoma aurantium*). A medium with ammonium was more effective. The authors noted the negative effect of the nitrate form of nitrogen and maleic acid on mycelial growth and determined the effective concentrations of glucose (20 g/L) and diammonium hydroorthophosphate (5–10 g/L), with an increase in the dose of these resins harming mycelial growth.

Daza et al. [47] found that the addition of albumin and glucose to the medium increased the intensity of mycelial dry matter accumulation and confirmed the positive role of ammonium in the cultivation of pure cultures of *Amanita caesarea* (Scop. Fr.) Pers. The authors determined the necessary acidity of the medium (pH 6–7) and the range of effective temperatures (24–28 °C). Bukhalo et al. [93] obtained similar results for the cultivation regime, with the range of optimal temperatures being 25–28 °C and pH of 5.5–6.0.

It is known from the literature that the energy of laser light stimulates the intensity of the transmembrane electrochemical gradient of protons in the mitochondria and cell proliferation and also causes morphological changes in cells and organisms [94]. A previous study [95] showed the positive effect of different types of Helium-Neon (He-Ne) and Argon (Ar) laser light (λ = 632.8 nm and 514 nm, respectively) on the growth of *H. mesophaeum* mycelia in pure culture. The authors concluded that the dynamics of cellular bioenergetic processes increase under the influence of laser light of a specific wavelength. On the third day of treatment, a significant increase in mycelium diameter was observed in the culture. They found that the growth rates of the mycelium were highest after exposure to a He-Ne laser (1 × 60 s), a cAr laser (2 × 60 s), and a He-Ne laser for 3 × 30. Container-grown *Pinus sylvestris* seedlings inoculated with an aqueous mycelial suspension of *H. mesophaeum* (previously irradiated with different types of laser light) showed a higher percentage of mycorrhizal associations on pine roots after 3 months (34.3% after He-Ne laser irradiation and after Ar laser irradiation) compared to the control group with untreated fungi. However, the fungus-infected seedlings were smaller than the seedlings in the control group. Overall, the laser light stimulated the mycelial growth of *H. mesophaeum* in pure culture and promoted the development of mycorrhiza in pine seedlings [95].

The positive role of chelators (citric acid, acetylacetone, HEPES), pH buffers, and nutrients on the growth of mycelium of *Lyophyllum shimeji*, *Rhizopogon rubescens, Suillus bovinus*, and *Tricholoma matsutake* was discussed in the work of Ohta [96].

The results of studies on the biomass of *Agaricus bisporus* and *I. badia* cultivated on a liquid medium with the addition of anthranilic acid and serine have shown that the concentration of the additive added to the medium is important for maximizing the biomass yield [40].

Minakov et al. [89] suggested that the optimal culture medium for the selection and growth of most isolates is agarized 4% (Balling scale) beer wort with the addition of thiamine at a concentration of 0.2 mg/mL. The authors found that the beginning of the process of overgrowth of the environment with mycelium depends on the type of fungus, a process that can take 3–4 weeks or longer.

Nikitina et al. [97] compared the growth of pure cultures of edible fungi on organic and inorganic agarized and liquid media and came to the conclusion that the best medium for the growth of mycelium is one prepared from wheat flour with the addition of oak sawdust extract. According to the authors, the growth of mycelium is greater in a liquid medium.

The positive effect of new-generation growth stimulants (Fumar and Biohumat) on the stage of teleomorph formation was confirmed in the studies of Kuznetsova [98]. The use of traditional growth stimulants (heteroauxin and gibberellin) was not effective at all concentrations. At a concentration of 10–3%, there was no significant positive effect, but at 10–4%, there was inhibition of mycelium development at almost all stages of morphogenesis and inhibition of mycelium development in the linear growth phase. Gibberellin had a stimulating effect on the development and density of the mycelium, but at the stage of teleomorph formation, the indicators did not always reliably differ from those of the control [99].

The inhibitory effect of pesticides and fungicides added to agar medium on mycelial growth in pure cultures of 64 ectomycorrhizal fungi from boreal forest trees has been demonstrated, with pesticides inhibiting the growth of different *Suillus* strains, and fungicides generally being more toxic to ectomycorrhizal fungi than herbicides. Knowledge of the pesticide tolerance of ectomycorrhizal fungi may be useful in deciding on their use in forest nurseries and forested fields [100].

The investigation of the influence of calcium orthophosphate (${\mathrm{Ca}}_{3}$(${\mathrm{PO}}_{4}$${)}_{2}$) and pure minerals, such as apatite, potassium feldspar, pink calcite marble, and quartz, on the regulation of the acid balance in the nutrient medium and the accumulation of dry matter showed that mycelium grown in the medium with the addition of marble had the highest density (*p* = 0.012), whereas that grown in the medium with the addition of potassium feldspar had the lowest density [101].

**Figure 1:**
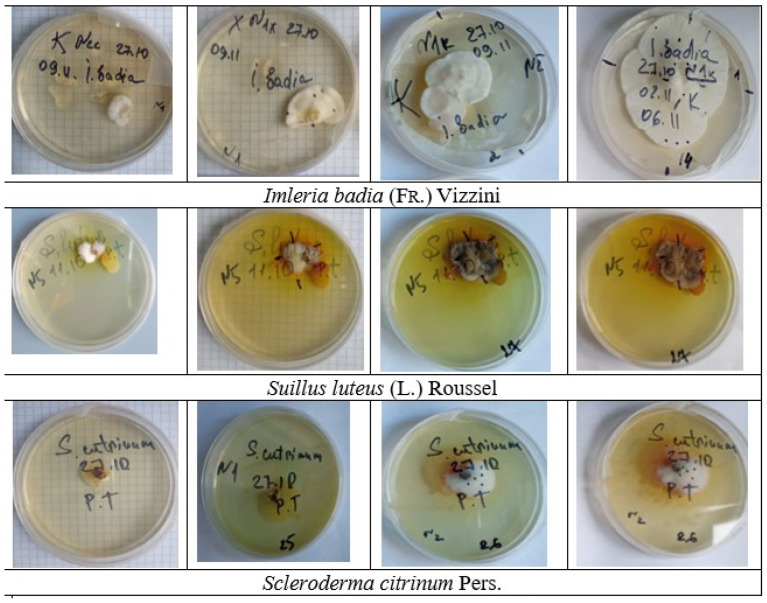
Dynamics of germination of mycelium of mycorrhizal fungi *Imleria badia* (Fr.) Vizini, 2014; *Suillus luteus* (L.) Rousel, 1796; and *Scleroderma citrinum* Pers. 1801 on an agar medium in vitro (photo V.D.). The agarized nutrient medium was prepared according to the Pachlewski [25] method. The cultures were aged for 10, 15, 20, and 30 days.

Kuznetsova and Vlasenko [102] showed that the growth of mycelium in an agarized substrate can be stimulated by the addition of vegetable oils (Figure 1). Sunflower, olive, and linseed oil were added to the culture medium at concentrations of 1 and 5%. The addition of sunflower oil to the culture medium resulted in an increase in radial mycelial growth of 43.5% compared to the control, whereas olive oil showed an increase of 39.1%. The addition of linseed oil did not lead to an increase in the mycelial development rate.

## 6. Peculiarities of the Mycorrhizal Structure and Its Influence on the Growth and Development of *Pinus* Species

The negative experience of reforestation of soils where no tree species previously grew indicates the need to artificially inoculate the roots of tree species with mycorrhizal fungi [103] Many gymnosperms, including the *Pinaceae* genera, are characterized by ecto- and ectendomycorrhizae. Such mycorrhizae are formed by fungi belonging to the classes *Basidiomycetes, Ascomycetes*, and *Zygomycetes*, which cover the laterally thickened roots with a dense network (mantle).

*Ectendomycorrhiza* is less researched, although it is similar to ectomycorrhiza, but differs from it by the presence of hyphae in the center of the root cells. Ectomycorrhizas do not penetrate the root cells with hyphae; instead, the presence of septate mycelium is observed between them [104]. Scientists note that the intensity of mycorrhizal formation depends on the quality of the forest vegetation [105]. Agerer [106] gave a comprehensive historical overview of the characteristics of ectomycorrhiza.

The morphological characteristics of the mycorrhizal form are related to the fungal species. Most species forming mycorrhizas of *Pinus sylvestris* are simple and dichotomously forked mycorrhizas with a small proportion of coralloid and tuberous forms. Simple mycorrhizal forms develop in species such as *Amanita rubescens, Cortinarius mucosus, C. vibratilis, Hygrophorus hypothejus, Tricholoma flavovirens, T. portentosum*, and *T. Terreum*, dichotomously forked species that often transform into coral-like formations; all species of the genus *Suillus*; and *Rhizopogon luteolas, Tricholoma imbricatum, Lactarius uridus*, and *Amanita muscaria*, from which tuberous forms have also developed. Young ectomycorrhizae are almost always white and can later become cream, beige, or brown, with the shades varying depending on the fungus species that form them [43]. Ectomycorrhizae of *Cenococcum graniforme* and *Mycelium radicis* atrovirens form a black mycelium [107]. *Ectomycorrhiza* with a smooth or only slightly hairy coat is observed in fungi of the genus *Lactarius* and *Lacoaria laccata, Amanita muscaria, Russula adusta*, and *Tuber albidum*, regardless of the environmental conditions. Other fungi produce various hyphae in the form of ends, rhizomorphs, mycelial veils, or bristles on their surface. Fungal species with high growth dynamics produce more auxins, which is particularly noticeable in fungi of the genus *Suillus* [25]. Mycorrhizal fungi belonging to the genera *Laccaria, Pisolithus, Amanita, Rhizopogon, Paxillus*, and *Hebelota* also synthesize auxins [61]. Compounds with cytokinin activity have been found in fungi of the genera *Paxillus, Rhizopogon*, and *Suillus* [108].

Research conducted in Ukraine has demonstrated the positive effect of the mycorrhizal fungi *Suillus luteus* and *Amanita muscaria* on the survival of pine seedlings planted in a 40-year-old pine stand after a forest fire. Scientists have found that the mycorrhization of seedlings before planting, regardless of the processing method, contributes to the maintenance and growth of the plants [29].

Attempts to introduce Korean cedar pine to Latvia were unsuccessful until semi-decomposed sediment from the undergrowth of this species was brought to the arboretum nursery, which contributed to the mycorrhization of the soil. The Austrian mycologist Göbl [109] proposed a gradation of the growth conditions of the European cedar pine according to the degree of mycorrhization of the roots. He believed that species with white, yellow, and gray mycorrhiza with stronger branching and multi-layered coverage were characterized by better growth. Poor conditions were characterized by dark mycorrhizae with weak branching and small-layered coverage [109].

Studies by Pachlewski and Pachlewska [25] on different types of mycorrhizal fungi in the root system of pine trees showed that the direction and intensity of mycorrhization are similar within certain genera. First and foremost is the genus *Suillus*. Species of this genus quickly form their first ectomycorrhizas; already 1.5 months after inoculation, a considerable amount of mycorrhizae from fungi of this genus can be seen in the root system of pine trees. It is important to protect the roots of the seedlings while they are still in the nursery. The *Rhisopogon* genus is characterized by similar properties. The duration of the formation of the first ectomycorrhiza for the genera *Lactarius* and *Russula* was only determined after 4 months from the time of inoculation. The formation of the first ectomycorrhiza in species from the genus *Amanita* takes 1–2 months from the time of inoculation, but the quantitative occurrence of ectomycorrhizas and their distribution in the root system is similar to *Lactarius, Russula*, and *Tuber*. The genera *Cortinarius, Hebeloma*, and *Triholoma* are the most numerous in ECM pines, although the formation of the first ectomycorrhizas was observed only after quite a long time in some of their species. They also differed significantly in the number and distribution area of mycorrhizae in the root system. The range of indicators of the intensity of ectomycorrhizal formation by individual fungal species was very wide, ranging from 8 to 450 ectomycorrhizae per seedling, which corresponded to 2 to 90% of the total number of short roots per seedling.

Richter and Bruhn [32] compared the survival of *Pinus tabuliformis* and *Pinus koraiensis* grown with mycelium/agar suspensions of three mycorrhizal fungi (*Laccaria bicolor, Scleroderma citrinum*, and an unidentified *Basidiomycete*) and another fungus (*Cantharellula umbonata*), which they hypothesized to be mycorrhizal. Saplings inoculated with *L. bicolor* were characterized by a higher survival rate, with the Chinese pine outperforming the Korean pine in both aspects (21% and 19%, respectively).

Artificial mycorrhizal seedlings were shown to have a higher assimilation rate, a higher shoot-to-root ratio, and a lower shoot growth rate compared to non-mycorrhizal seedlings. The authors hypothesized that the mycelium of *S. citrinum* retains a considerable proportion (32%) of the nitrogen supplied to the plants, significantly reducing its uptake by the host plants [69]. It is very likely that the plants first invest in root development, which may increase by up to 1000-fold. Then, a better water supply increases the photosynthetic capacity and more assimilates are sent into shoot development. The effect of mycorrhiza thus shifts from the first years of growth to the later benefits that such a symbiosis offers for both components.

The artificial inoculation of pine seedlings has shown [16,110] that mycorrhizae can harm the growth of the host plant. This property can be explained by the fact that carbon and nitrogen are taken up by mycorrhizae and stored underground [111].

Machon et al. [112] investigated the effect of the ectomycorrhizal fungus *Laccaria laccata* on *Pinus pinea* seeds infected with *Fusarium verticillioides* and *F. oxysporum*. The results of inoculation with *Laccaria laccata* were not noticeable until 18 weeks after sowing, while seed germination was not significantly affected by inoculation. *L. laccata* reduced the frequency of seed washout immediately after germination, but the differences compared to the control were only significant in the treatment with *F. oxysporum*. Inoculation with *L. laccata* did not significantly reduce the virulence of *Fusarium species*, the percentage of mycorrhization did not reach a significant level, and the amount of mycorrhizal fungus was not sufficient for effective protection.

Sirenko et al. [113] investigated mycorrhiza on the roots of 1-year-old pine seedlings obtained from the nurseries of state-owned companies and on selected seedlings of natural origin collected from an 85-year-old pine plantation. The intensity of mycorrhization of the pine seedlings in culture was almost twice as low as in nature (40–45% and 75–80%, respectively), and the mycorrhizal density was 76% lower. The mycorrhiza on pine seedlings of natural origin was characterized by a greater diversity of fouling species (four to two) and a greater thickness of the mycorrhizal layer, as well as a lower depth of penetration of the mycorrhizal infection into the root tissue. The color of the mycorrhizal ends of the natural regeneration of pines from natural stands varied from white-yellow to dark brown and black, and in cultivated stands, it was brownish-brown.

Salgado et al. [114] investigated the presence and quantity of viable mycorrhizal inocula produced by *P. ponderosa* plantations in the Patagonian steppe and evaluated their dispersal ability. *P. ponderosa* plantations were an effective source of mycorrhizal inoculum that established and persisted in neighboring areas but had a low dispersal rate that decreased sharply with the distance from the plantation.

Aleksandrowicz-Trzcinska and Buraczyk [115] investigated pine cultures planted on opencast lignite mines in Bełchatów. For the cultures, planting material with an open root system (ORS) was used, which was grown in a nursery (with soil filling) and a greenhouse, as well as seedlings with a closed root system (CRS), which were grown in containers with and without the addition of the mycorrhizal fungus *Hebeloma crustuliniforme*. Three years after planting, six morphotypes of mycorrhizal fungi were found on the roots of the seedlings: ectendomycorrhizae (brown, with light-coloured tips, thin smooth coat, branched with white filaments, simple and dichotomous forms); *Suillus* (mycelium ranging from light to dark brown, with white, gray, or gray-brown mycelium standing out from the mantle, occurring rarely or very abundantly, adherent to the tips of the mycorrhiza, with thick, well-developed rhizomes, either white or the same color as the mycorrhiza, occurring singly, with dichotomous and coralloid forms); *Hebeloma* (light-colored, elongated mycorrhiza with fairly abundant white mycelium, sometimes in the form of rare crystals, with single and dichotomously branched forms); *Thelephora* (mycorrhiza with a smooth mantle); black mycorrhiza (with black mycelium, ranging from very abundant to single hyphae, emerging from the mantle); and light-coloured mycorrhiza (mycorrhiza with white-pink mycelium, fluffy and very abundant, with abundant, thick rhizomorphs frequently releasing mycelium, predominated by coralloid and tufted mycorrhiza) [115]. The best growth parameters (height and diameter of the root collar) and grafting possibilities were offered by the cultures obtained from seedlings with an ORS from the nursery. They exhibited all six morphotypes of mycorrhiza. In root samples from seedlings with a CRS without mycorrhiza, black-type mycorrhiza was detected [115].

Buraczyk et al. [116] concluded that the technology used to grow the planting material in the nursery has a greater influence on the development of pine growth parameters compared to soil fertility and soil moisture. Substrate moisture in the range of 30–50% did not affect the biometric parameters of Scots pine, regardless of the type of planting material (mycorrhiza and non-mycorrhiza) and substrate (forest and post-agro soil) used. The results showed that pines mycorrhizalized with the fungus *H. crustuliniforme* had significantly lower parameters than non-mycorrhizalized pines and that the type of substrate had no influence on the growth of the seedlings.

The results of a study by Andreeva et al. [117] showed the opposite. According to their data, the types of soil conditions in the system have a significant influence on the growth of one-year-old *Pinus sylvestris* seedlings and the mycorrhization of their root system.

**Figure 2:**
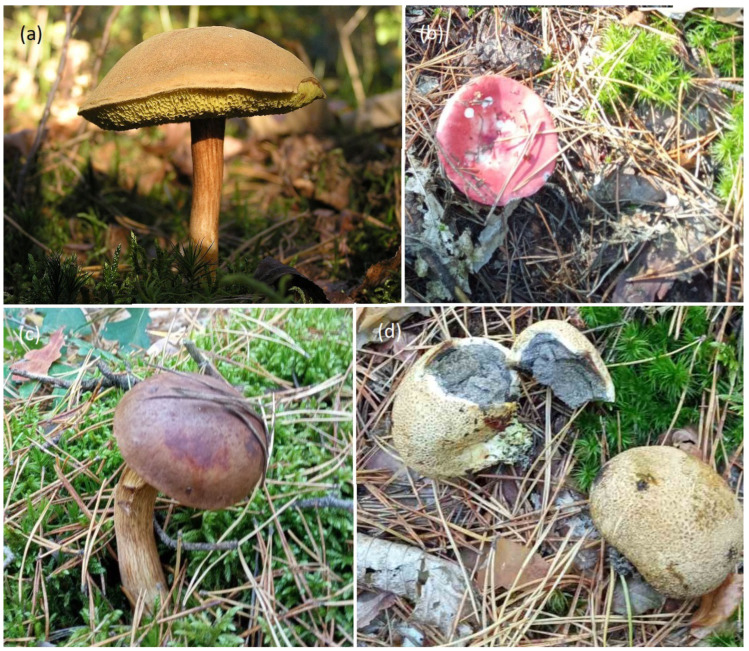
Selected examples of mycorrhizal fungi in a pine forest: fruiting bodies of *Xerocomus subtomentosus* (**a**), *Russula paludosa* (**b**), *Imleria badia* (**c**), and *Scleroderma citrinum* (**d**) in the litter of a pine forest (photo V.D.).

Hilszczańska [19,20,95,118,119,120,121] studied in detail the structure of pine mycorrhiza, the properties of mycorrhizal fungi, and the influence of substrate moisture on the mycorrhization of plants in forest nurseries. According to the data obtained, seedlings with a CRS were characterized by a lower number of natural mycorrhizae than seedlings with an ORS. At the same time, the pine seedlings with mycorrhizal roots adapted better to the environment compared to the control plants but were inferior to them in terms of growth parameters (length of the above-ground part). The work also confirmed the positive effect of soil moisture on mycorrhizal biodiversity. Other studies have also confirmed that mycorrhization is sometimes accompanied by a slight slowdown in the growth of plant shoots [69], and this applies not only to *Scleroderma* but also to other types of mycorrhizal fungi [121] (Figure 2).

**Figure 3:**
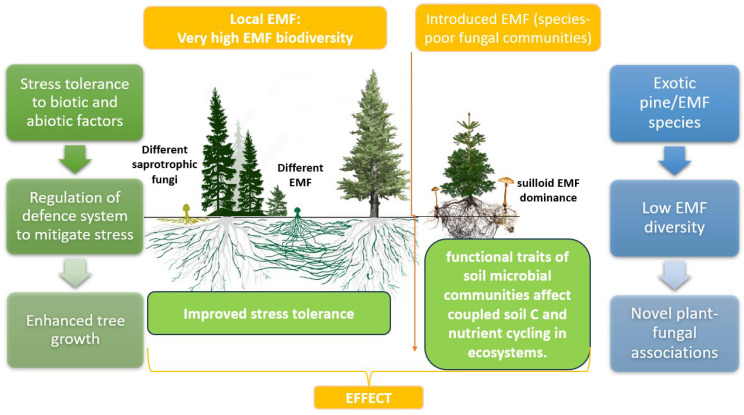
Mycorrhizal networks in plant–fungi symbiosis.

The introduction of exotic pines into plant plantations, often accompanied by novel ectomycorrhizal fungi or the application of non-local ectomycorrhizal fungi, may have significant ecological implications. This symbiotic relationship between pines and specific ectomycorrhizal fungal lineages, particularly Suilloid genera such as Suillus and Rhizopogon, can contribute to the invasion of pines into native habitats. These fungi, adapted for early succession in their native range, facilitate the establishment and spread of pines outside plantations [46,122]. In this case, pines can successfully invade with the exclusive presence of Suilloid species, showcasing their unique traits, including abundant spore production and efficient dispersion. These fungi, constituting a minor percentage in their native range, play a crucial role in pine invasions. The simplified fungal communities in pine plantations, dominated by a small number of fruiting species like Suillus luteus, result in exceptionally high fruiting biomass compared to native pine habitats (Figure 3).

The mechanisms behind this dominance, whether due to enemy release, reduced fungal diversity, plastic responses, or rapid evolution, remain untested [122]. Nevertheless, the introduction of low-diversity ectomycorrhizal fungal suites alongside pines can have cascading consequences, thereby influencing population dynamics, invasion dynamics, and ecosystem functions. Large-scale natural experiments involving plant–ectomycorrhizal fungal co-introductions provide valuable opportunities to explore these complex interactions and their ecological consequences.

## 7. Effects of Climate Change on Mycorrhizal Fungi

Climate change can have an impact on mycorrhizal fungi, as it does on all other living organisms. These fungi are expected to play an important role in ecosystems in response to global warming [123,124,125]. The overall carbon distribution and functioning of the forest ecosystem are highly dependent on the interaction between host trees and mycorrhizal fungi, especially in the context of climate change [126]. Hawkins [127] estimated that about 13 Gt ${\mathrm{CO}}_{2}$ are annually translocated to mycorrhizal fungi, which represent the most important carbon pool, and this fact necessitates the inclusion of mycorrhizal fungi in future climate change models.

Bennett and Classen [128] documented that mycorrhizal fungi are more affected by changes in temperature and precipitation than by increased carbon dioxide in the context of climate change. Responses to climate change depend largely on the type of mycorrhizal fungi, the host species with which they live in symbiosis, the soil type, and the geographical location in which they occur. Ectomycorrhizal fungi appear to be more variable in their response to climate change compared to arbuscular mycorrhizal fungi [128], whereas arbuscular mycorrhizal fungi are less cold-tolerant compared to other types of mycorrhizal fungi [129].

In terms of host species, the growth and fruiting of mycorrhizal fungi associated with evergreen trees are more delayed at higher temperatures compared to deciduous trees [130]. In addition, a greater diversity of mycorrhizae is expected at higher temperatures in forests than on arable land [131]. A higher richness of mycorrhizae in Vertisol soils compared to chernozem soils [132] or in sandy soils compared to clay soils [133] could additionally be influenced by extreme temperatures or precipitation [134].

In the face of overall climate change, mycorrhizal fungi may have the potential to contribute to the restoration of temperate and boreal forests and the maintenance of sustainable forest ecosystems under the stressors of global change [135]. Compared to non-mycorrhizal plants, plants in symbiosis with mycorrhiza have a greater tolerance to abiotic stressors (temperatures, ${\mathrm{CO}}_{2}$, drought, and salinity) due to the stimulation of the production of molecules to protect against cell damage; morphological, physiological, and molecular mechanisms; and the transport of pollutants [136,137,138,139,140,141,142,143]. In addition, mycorrhizal fungi could play an important role in promoting the evolutionary adaptations of plants to global warming [144].

Mycorrhizae have the potential to act as symbionts to help their hosts combat climate change by successfully maintaining their diversity and fertility in stressful situations common in the context of global changes such as increased ${\mathrm{CO}}_{2}$, extreme temperatures and soils, and precipitation variability [145].

In this context, it is of utmost importance to maintain native and stress-tolerant strains of mycorrhizal fungi to improve the performance and yield of their ’faithful’ plant hosts.

For this reason, research on mycorrhizal fungi should be intensified to increase knowledge of them and utilize them as a natural, safe, and environmentally friendly means of maintaining the stability of forest ecosystems to address climate change.

## 8. Role of Mycorrhiza in the Control of Pathogens

The soil microflora is very diverse and contains all forms of microorganisms that exist on Earth: bacteria, viruses, actinomycetes, yeasts, fungi, and protozoa [145]. The establishment of plantations on disturbed and degraded soils, where the forest microflora is absent and microorganisms that are not characteristic of these soils are present, affects the productivity and resistance of forest trees to damage by pathogenic fungi [146]. The search for measures that promote better growth and development of forest tree species planted on disturbed and degraded soils is an extremely urgent task under the conditions of climate change and the consequences of hostilities. Studies by Zak [147] showed that artificial warming of the forest soil during a growing season, with a temperature increase of 1 °C, kills the roots and leads to the death of 6–19% of the roots. An increase of 2 °C kills almost 60% of the roots, and an increase of 6–7 °C kills almost all roots.

The basis for measures to restore soil biodiversity and protect plants from pathogens can be the use of soil microorganisms, including mycorrhizal fungi. Scientists in various countries are conducting research in this direction, but the mechanisms of such protection have not yet been sufficiently investigated. There is ample evidence that mycorrhizae promote growth and are often essential for survival. This fact is clearly illustrated by numerous failures in attempts to establish trees in previously treeless areas [90,148].

An evaluation of the antibiotic activity of mycorrhizal fungi in the rhizosphere was conducted in [149]. The study concluded that the organisms of the rhizosphere compete with the pathogenic fungi for the attractants secreted by the root and form an antibiotic barrier. In the author’s opinion, the rhizosphere can serve as a biological filter that filters out and neutralizes soil toxins. The selectivity of the effect of mycorrhizal fungi has been confirmed in the studies of many scientists [150,151]. According to these authors, the effect of mycorrhizal fungi on the plant and the environment can vary depending on the type of plant, its genotype, its stage of development, its growth rate, and the type of soil. Some symbionts create a more effective biological barrier; their protection can extend not only to the roots but also to other parts of the tree. Some fungi can secrete antibiotic substances both in the rhizosphere and in the tissue of the roots.

According to research [152,153], mycorrhizal fungi compete with pathogenic fungi for carbohydrates and other metabolic products in the rhizosphere, form mechanical barriers in the form of covers that prevent pathogens from entering the root tissue, and secrete antibiotics and stimulate root cells to produce chemical inhibitors. All these mechanisms work together and it is quite difficult to figure out the role of each one.

Research by Zhdanyuk [154] proved that mycorrhiza improves the resistance of plants to pests and pathogens by stimulating profound changes in their metabolism. The author found that, in contrast to non-mycorrhizal plants, mycorrhizal plants secrete more jasmonate hormones and salicylic acid, which are key components of their defense system.

To penetrate the root tissue, the pathogen must physically and chemically penetrate the fungal barrier formed by the mycorrhizal fungi. In this case, the thick shell of their mycelium offers the plants better protection compared to a thin shell. Zak’s research [147] confirmed that the effectiveness of a thick mycorrhiza with a thickness of several micrometers and close-fitting hyphae is greater than a thin one.

Most short roots are mycorrhizal, whereas long roots are rarely mycorrhizal, probably due to rapid growth [155].

Chakravarty and Unestam [156] investigated the effect of *Laccaria laccata*, *Hebeloma crustuliniforme*, *Pisolithus tinctorius* mycorrhizae, and a mixture of natural mycorrhizae on the resistance of *Pinus sylvestris* seedlings inoculated with *Fusarium moniliforme* and *Rhizoctonia solani*. In the presence of mycorrhizal fungi, the severity of the disease and plant death caused by these pathogens was drastically reduced even before mycorrhizae had formed. A long-lasting (about one year) positive protective effect was observed in all variants with the use of mycorrhizal fungi, except for *Hebeloma*, whereas plants inoculated with the *Hebeloma* species were protected for only about 3 months. The authors proved that not all species of mycorrhizal fungi have antagonistic properties.

The formation of pseudomycorrhiza is often the cause of root diseases in old trees [157,158,159].

According to Zak [147], the intensity of metabolic processes in trees decreases with age. Young, vigorous trees (up to 20 years old) are less affected by pathogens, replace lost tissue more quickly, and restore normal growth. The growing conditions also have a significant influence on the development of the disease and the time at which the external symptoms of the disease appear.

Studies by Radic et al. [160] and Neethu et al. [161] confirmed the ability of sporophore extracts of many basidiomycetes, which include mycorrhizal fungi, to inhibit bacterial growth. A negative result was obtained when *Amanita muscaria* was tested as an antagonist against *Phytophthora cinnamomi* and *Cylindrocladiuni scoparium*, whereas *Boletus* sp. inhibited the pathogens [147,162,163]. Similar tests were carried out by the author with isolates of basidiomycetes from pine mycorrhizae. Some of them showed antagonism toward *Cenococcum graniforme*.

Santoro and Casida [164] investigated the ability of the mycorrhizal fungi *Amanita muscaria*, *Boletus bicolor* (*B. rubellus*), and *B. luteus* (*Suillus luteus*) to produce antibiotics, which may be a factor for plant survival in nature.

A screening test was performed with 85 isolates of soil fungi, some of which were mycorrhizal fungi, to determine their ability to show antagonistic properties against the root rot fungus *Fomes annosus*. The pathogen-induced disease was most strongly suppressed in variants inoculated with the species *Boletus bovinus*, *B. variegatus*, and *Monotropa hypopitys* [165].

The problems associated with increasing the stability of plantations and their conservation in the face of climate change are prompting the search for new ways to increase the adaptive capacity of plants, which depends on the persistence of the organism and the introduced populations as a whole. Addressing these issues will make it possible to solve several problems, not only in the introduction of plants but also in agriculture, forestry, and urban greening.

It is a generally recognized fact that mycorrhiza promotes the growth of trees and is necessary for their survival. However, the mechanisms of interaction between plants and mycorrhiza are still not clear, and the physiology of mycorrhizal fungi still requires considerable research. The antagonistic properties of mycorrhizal fungi and their ability to produce antibiotics, as well as their role in promoting the survival of plants under stress conditions, deserve special attention. The forest floor is a complex and variable environment that is difficult to study. Therefore, laboratory studies and the cultivation of pure cultures can be effective in further exploring the properties of mycorrhizal fungi.

## 9. Conclusions

To summarize, the role of mycorrhizal fungi in supporting the growth and survival of trees, especially in reforestation measures, is evident from various studies and observations. Negative experiences with reforestation on soils without previous tree species emphasize the importance of artificially inoculating tree roots with mycorrhizal fungi.

The diversity of mycorrhizal forms, such as ecto- and ectendomycorrhizae, and their morphological characteristics contribute to the symbiotic relationship with different tree species. In particular, certain genera such as *Suillus, Amanita*, and *Rhizopogon* have been identified for their positive effects on the survival of pine seedlings, highlighting the potential for targeted mycorrhizal inoculation in reforestation projects [166].

However, it is important to consider the species-specific dynamics of mycorrhizal formation. For example, the genus *Suillus* shows rapid ectomycorrhizal formation in pine trees, which emphasizes the importance of protecting seedling roots during the early stages in the nursery [167].

While mycorrhizal symbiosis generally increases plant resilience to abiotic stress factors such as temperature fluctuations and soil conditions, some studies point to potential drawbacks. The artificial inoculation of pine seedlings indicated that mycorrhizae can impair the growth of the host plant by absorbing and storing carbon and nitrogen underground [168].

The protective function of mycorrhizal fungi against pathogens is significant. Research highlights their potential to inhibit the growth of pathogenic fungi, form antibiotic barriers, and stimulate plant defense mechanisms. This defense capability contributes to the overall health and survival of trees, especially in disturbed and degraded soils [169].

In the context of climate change, mycorrhizal fungi are expected to play a crucial role in the restoration and conservation of temperate and boreal forests. The potential of mycorrhizae to mitigate the effects of global changes, such as increased ${\mathrm{CO}}_{2}$, extreme temperatures, and precipitation variability, emphasizes the need for further research and conservation measures.

Given the challenges posed by climate change, understanding the complex interactions between trees and mycorrhizal fungi is becoming increasingly important. The conservation of native and stress-tolerant strains of mycorrhizal fungi is emphasized as a natural, safe, and environmentally friendly strategy to improve the stability of forest ecosystems and combat the effects of climate change. More intensive research into mycorrhizal fungi will provide valuable insights for their use in sustainable forest management.

## Data Availability

Not applicable.
